# Vindication of the Pre-Eminent Efficacy of Manual Operations in the Cure of Rheumatic and Nervous Diseases, against the Malignant and Unprincipled Attempts of Certain Members of the Medical Profession to Decry and Obstruct the Practice

**Published:** 1834-10-01

**Authors:** 


*Vindication of the pre-eminent Efficacy of Manual Operations in the Cure of Rheumatic and Nervous Diseases, against the malignant and unprincipled Attempts of certain Members of the Medical Profession to decry and obstruct the Practice.* By William Balfour, m.d., l.r.c.s.—Edinburgh, 1834. 8vo. pp. 24.

Dr. Balfour is certainly the most ill-used doctor that we have met with for an age. Here has he been twenty years knocking rheumatism out of the world with an assiduity beyond all praise; and yet a number of persons, in the garb of men, but who take very little pains to conceal their cloven feet, go about, and endeavour to obstruct his practice: aye, *obstruct*, for that is the word. The devilries of the anti-percussors are six in number.

"*In the first place.* Some time ago, I made a perfect cure of lameness in a lady, of twenty-four years' duration, eighteen of which she was on crutches. Conceiving it would be for the benefit of many a distressed person to make the case generally known, I had it printed, and advertised it to be had gratis at my house, and at two separate booksellers' shops. One of these booksellers distributed all the copies I sent him, the other gave out very few. When I asked the reason, he said that '*several* medical men in the city (naming some of them,) had called on him, and stated that, if he gave out a single copy of my pamphlet, they would withdraw their custom and patronage from him!'

"Now, in conduct so nefarious, illegal, and unjust, whether did malignity or meanness predominate? They stand, I think, in this relation to each other: the malice of the ignoble individuals in question was of such virulence, as to make them lose sight of all self-respect, in their endeavours to crush me; for no man, not transported with the most outrageous jealousy and envy, could ever lower himself so far as to attempt, in so mean, wicked, dastardly, and covert a manner, to mar the success and obstruct the usefulness of another, who never crossed his path, and never was likely to do so.

"There was no allusion to these wretched imbeciles in the pamphlet they thus endeavoured to suppress: they never received provocation at my hands, of any kind or degree; I never spoke to a single individual of them in all my life, except one, who has seen my practice, and declared it, in a letter of his which I hold, to be 'efficient, scientific, and a blessing to suffering humanity.' This person is a prominent advocate for *the liberty of the press:* nevertheless, I detected him in secretly attempting to suppress the pamphlet referred to, which pamphlet had no other object, and could have no other object, than the good of mankind. Whence, then, could such gratuitous malice proceed? The answer is obvious: it arose from envy at my daily curing cases which had been given up for many years as hopeless, and my thereby obtaining the patronage of the public, while my unprincipled opponents and persecutors are consuming their days in idleness and obscurity. That is 'the head and front of my offending,' the gall and wormwood which turn their stomach." (P. 4.)

Yet some would alledge that these pamphlet-swampers, or, as our author calls them, "wretched imbeciles,"—these lovers of rheumatism, who instantly ossified one of his channels of circulation, and deprived half Edinburgh of its book, were really blessings in disguise. The sturdiest physician, sound in wind and limb, can scarcely get through a practice of fourteen hours a day. What then would have been our author's fate, with all the Old and all the New Town knocking at his portals, had the obstructors not happily come in the way, and reduced the eagerness of the Edinburgh rheumatics within bearable limits? One of the "wretched imbeciles" we hold to have been a bungler. We mean the one who, after declaring Dr. Balfour's practice to be "efficient, scientific, and a blessing to suffering humanity," was detected "in secretly attempting to suppress the pamphlet referred to." He ought to have known that libricide is a capital crime in authors' eyes, and should have been careful to smother the pamphlet in twilight only, by winks, nods, and oracular insinuations. Yet he was most probably detected with large bundles of the *perfect cure of lameness* in his pocket, with a string around their necks, and about to consign them to some of those murky repositories, whence pamphlets rarely emerge—save to wrap up tooth-brushes, light cigars, or be eternally shaved upon! We perfectly agree, too, with our author, that this knavish obstructor must be a most hypocritical champion of "the liberty of the press;" unless indeed he means by this phrase the liberty of pressing the struggling pamphlets into the lowest abysses of his pockets. As no one, however, unites every fault in his own person, it must be allowed that the knave just mentioned, who was so fond forsooth of the liberty of the press, was not one of those that Milton talks of,

> "Who bawl for freedom in their senseless mood,
> And licence mean, when they cry liberty;"

no, indeed; such liberty of the press as he allowed might satisfy the autocrat of all the Russias: print what you please, only don't publish; if you even give away a copy, not a stiver shall you ever pouch again to the end of time. Nevertheless, we are surprised at Dr. Balfour's declaring that the wretched imbeciles had received no provocation at his hands. Now, we think they had received a most mortal one. Dr. B. had omitted all mention of them in his pamphlet; and these idle doctors, these men without patients and without patience, possessing no talent but that of book-stifling, were excluded from their sole hope of immortality; for what chance have flies of preservation, but by being included in amber?

But we ask pardon of our readers for these comparatively trifling remarks, by which we have detained them so long from the instructive pages of our author. Hear his second dirge.

"*In the second place.* It is no uncommon occurrence for me to receive patients from afar, who have for many months, and some of them for many years, been subjected to great pain and lameness, and consequent impaired health, without having obtained the least alleviation of their complaints from medicine. When I ask such patients, why they did not apply to me sooner? the uniform reply is, 'We were prevented by our doctors, who assured us there was nothing in your practice, that it would never cure any body, and that, at any rate, it was not applicable in our cases; but, hearing of other patients whom you had cured, in circumstances similar to our own, we came off at last, without our doctors' leave.' I shall here give two or three cases, in illustration.

"1st. A young lady, in a northern county, came to complain of pain and weakness in her back, right thigh, and leg, without any known cause. In defiance of all her medical advisers could do, she continued to grow worse for eighteen months. A consultation was then held, when her case was decided to be hopeless. On it being suggested that I should be called in, her doctors deprecated the idea, assuring the patient and her friends that my practice was inapplicable in her case, and that it would certainly kill her. She came to me, notwithstanding; and returned home, in three weeks, perfectly well, and has continued so for upwards of ten years.

"Now it irresistibly follows, either that this young lady's doctors were wicked, unprincipled men, or that they were profoundly ignorant of the efficacy and safety of my practice. In charity, I shall assume the latter as the true alternative; and I then ask, how came they to pronounce a practice inapplicable, nay killing, about which they knew absolutely nothing? The lady never once complained under my hands." (P. 5.)

We deeply regret being compelled to abridge the marrowy sentences of our author, but we must make short work with the rest. The young lady just mentioned got home, and fostered a rheumatic insurrection among her townsfolk. She says, in a letter to our author, "The medical men here all wish you as high as Haman. You have put their rheumatic patients in a state of rebellion, and I shall do everything in my power to increase it." (P. 6.) This letter is dated in 1823, but our author does not state how the rebellion turned out; whether a detachment of patients fairly gave their doctors the slip, and hobbled off to Edinburgh, or whether they still groan (uncompressed) under the tyrannic sway of their professional tormentors.

We now come to our author's *in the third place.* It seems that some of the malignants keep a kind of cads, or liers-in-wait, whose business it is to fib against Dr. Balfour. "Not a person of consideration, lady or gentleman, comes to me from a distance, (and many such come,) but my professional opponents, and their infamous go-betweens, are immediately at work, to prejudice them against my practice; and there is no misrepresentation too gross, no falsehood too atrocious, for them to employ, to compass their end. This is no fancy; my information is from my patients themselves, most of whom soon penetrate the motives of their false and officious friends, and consequently treat them with merited contempt." (P. 8.)

The reasons of this hostility are two in number, being the number of the offences which our author confesses to have committed against science and society. "I have been guilty of no offence, that I am aware of, either against science or society, but two,—that of ascertaining a law of the animal economy, whereby a part, entirely separated from the living body for a length of time, can be reunited, and again become part of the system; and the method, which I am now vindicating, of curing rheumatic, nervous, and some other diseases, which prove incurable by any other means: delinquencies these, which, all the world knows, cannot be tolerated in any contemporary, by gentlemen in that grade of the profession who honour me with all the paltry hostility they can muster." (P. 8.) This frank avowal of his faults, on our author's part, is worthy of the highest praise, and can be paralleled only by the candour of those professors of humility who, as Lord Chesterfield informs us, were wont, in his time, to confess themselves guilty of all the cardinal virtues.

Again we say, Dr. Balfour is the most ill-used man in the world: let him cure nineteen patients, and no one breathes a word on the subject; the half-uttered praise expires on the lips of the few and frightened Balfourians. But let him fail only once, and all Edinburgh rings with it: the long-winded prosing of the pursy baillie, and the withering smile of the epigrammatic physician, find a common object in the unfortunate case. "If, out of twenty desperate, inveterate cases, which have long resisted every other mode of treatment, I cure nineteen, and fail in the twentieth, that single failure meets me at every corner, is bandied about in all directions, as conclusive evidence *against* my practice; but the nineteen are kept in the background,—are never once mentioned,—they form no evidence *in favour* of the same practice!"

Here we must conclude, and break off our review abruptly; for, though we could be well content to go on with this delicious little pamphlet for ever, we fear lest our sterner readers should complain of our devoting so large a space to so small a work. We therefore take leave of our author, assuring him that we envy him his power of curing nineteen desperate cases of rheumatism out of twenty; and though we, who are unambitious reviewers, if we possessed the power, might be content with a moderate practice of some 10,000*l.* per annum, we see no reason why Dr. Balfour should not take a loftier flight;—why he should not realize a fortune beyond the dreams of avarice, and, turning the tables on the "wretched imbeciles," become in turn their obstructor.

